# Adiposity and the risk of rheumatoid arthritis: a systematic review and meta-analysis of cohort studies

**DOI:** 10.1038/s41598-020-71676-6

**Published:** 2020-09-29

**Authors:** Tomoya Ohno, Dagfinn Aune, Alicia K. Heath

**Affiliations:** 1 Department of Epidemiology and Biostatistics, School of Public Health, Imperial College London, St. Mary’s Campus, Norfolk Place, Paddington, London, W2 1PG UK; 2 Department of Nutrition, Bjørknes University College, Oslo, Norway; 3 Department of Endocrinology, Morbid Obesity and Preventive Medicine, Oslo University Hospital, Oslo, Norway; 4 Present Address: Oncology Division, Novartis Pharma K.K., Tokyo, Japan

**Keywords:** Rheumatology, Risk factors

## Abstract

Several studies have investigated associations between overweight/obesity and risk of developing rheumatoid arthritis, however, the evidence is not entirely consistent, and previous meta-analyses mainly included case–control studies, which can be affected by various biases. We therefore conducted a systematic review and meta-analysis of cohort studies on adiposity and risk of rheumatoid arthritis. Relevant studies were identified by searching PubMed and Embase databases. Random effects models were used to estimate summary relative risks (RRs) and 95% confidence intervals (CIs) for rheumatoid arthritis in relation to different measures of adiposity. Thirteen cohort studies (10 publications) were included. The summary RR per 5 kg/m^2^ increase in body mass index (BMI) was 1.11 (95% CI 1.05–1.18, *I*^2^ = 50%), but the association was restricted to women (1.15, 95% CI 1.08–1.21, *I*^2^ = 17%) and not observed in men (0.89, 95% CI 0.73–1.09, *I*^2^ = 58%). The summary RR per 5 kg/m^2^ increment in BMI at age 18 years was 1.17 (95% CI 1.01–1.36, *I*^2^ = 26%, n = 3), and per 10 cm increase in waist circumference was 1.13 (95% CI 1.02–1.25, *I*^2^ = 44%, n = 2). Higher BMI in middle age, BMI at age 18 years, and waist circumference were associated with increased rheumatoid arthritis risk, suggesting adiposity could be targeted for primary prevention.

## Introduction

Rheumatoid arthritis is a systemic autoimmune disease characterized by an inflamed synovium which can cause pain, swelling, stiffness and deformity of multiple joints^1,2^. Globally an estimated 23.7 million people live with rheumatoid arthritis^3^. There is wide geographic variation in the prevalence rates of rheumatoid arthritis between countries, with rates ranging from 0.2 to 1.1% between regions, and higher rates in high-income countries compared to low- and middle-income countries^4,5^. For example, prevalence rates of 0–3 cases per 1,000 persons have been reported in some areas in Africa, while rates in Northern and Western Europe are around 4–11 cases per 1,000 inhabitants, and in North America between 9 and 11 cases per 1,000 inhabitants^4^. These geographical differences in the occurrence of rheumatoid arthritis may be attributed to various factors, including genetic factors, socioeconomic factors, access to health services, and lifestyle factors^6^.

Established or suspected risk factors for rheumatoid arthritis include age^7^, genetic predisposition (human leukocyte antigen (HLA)-DR4)^8^, smoking^9,10^, diet^10–12^, physical activity^13^, and obesity^14–24^. In addition, the global prevalence of rheumatoid arthritis has been reported to be approximately two times higher in women than in men^25^. These sex differences suggest that hormone-related factors or other sex-specific exposures may contribute to the development of rheumatoid arthritis^26^. Since body fat distribution differs for men and women, it is also possible that the differences in adiposity between men and women might, at least in part, account for the sex difference in rates of rheumatoid arthritis^27^.

Several epidemiological studies have suggested an increased risk of rheumatoid arthritis associated with overweight and obesity^14–19^, but other studies found no clear association^20,21^. Although several meta-analyses have found positive associations between body mass index (BMI) and risk of rheumatoid arthritis^22–24^, they were mainly based on case–control studies which could be affected by recall and selection biases and reverse causation^7^. In addition, these meta-analyses only examined BMI and did not consider other measures of adiposity which might be more pertinent for rheumatoid arthritis. A few studies have suggested positive associations for BMI in early adulthood with rheumatoid arthritis^15^, and other studies suggested that abdominal fatness may also be important^18,19^, however, these results have not previously been summarised in a meta-analysis. Clarifying whether abdominal adiposity is more relevant for rheumatoid arthritis than general adiposity could contribute towards a better understanding of the underlying mechanisms and more targeted interventions for prevention and treatment. Therefore, to provide a more comprehensive assessment, we conducted an updated systematic review and meta-analysis of cohort studies to clarify the strength and shape of the association between different measures of adiposity and the risk of developing rheumatoid arthritis.

## Methods

### Search strategy

An electronic literature search was conducted in Embase and PubMed databases to identify all eligible studies on the association between adiposity and the risk of rheumatoid arthritis that were published up to May 10th, 2019. The following search strategy was used in PubMed and a similar search was adapted for the Embase search: (“body mass index” or body mass index[MeSH] or BMI or overweight or overweight[MeSH] or obesity or obesity[MeSH] or anthropometry or anthropometry[MeSH] or fatness or “body fatness” or “abdominal fatness” or “abdominal obesity” or abdominal obesity[MeSH] or “waist circumference” or "hip circumference" or “waist-to-hip ratio” or waist-to-hip ratio[MeSH] or adiposity or adiposity[MeSH] or "weight gain" or weight gain[MeSH] or "weight change" or weight change[MeSH] or “weight loss” or weight loss[MeSH]) AND (“rheumatoid arthritis” or rheumatoid arthritis[MeSH]) AND ("case–control" or cohort or prospective or longitudinal or retrospective or "follow-up" or "cross-sectional" or "hazard ratio" or "hazard ratios" or "relative risk" or "relative risks" or "incidence rate ratio" or "incidence rate ratios" or "odds ratio" or "odds ratios" or incidence).

### Study selection

Records identified from the search strategy were screened based on the title and abstract in the first step, and then further assessed for eligibility based on the full text in the second step. Prospective and retrospective cohort studies, case-cohort studies, and nested case–control studies within cohort studies, which reported adjusted relative risk (RR) estimates (RRs, hazard ratios (HRs), incidence rate ratios, odds ratios (ORs)) and 95% confidence intervals (CIs) for rheumatoid arthritis for at least three or more categories of any measure of adiposity (e.g. BMI, waist circumference) or a risk estimate on a continuous scale were eligible for inclusion in this meta-analysis. Grey literature such as conference abstracts were excluded because they are unlikely to contain all the information required for dose–response analyses and for evaluation of study quality. Reviews, letters, comments, editorials, meta-analyses, meta-synthesis, prediction models, protocols, as well as epidemiological studies in patient populations were excluded. If there were duplicate publications from the same study population, the publication with the largest number of cases or with the most detailed information needed for dose–response meta-analyses was included. The initial screening of all references based on abstracts and titles was done by one author (TO) and a second author (DA) independently did the second part of the screening in duplicate.

### Data extraction

The following data were extracted from each publication: name of the first author, publication year, country, study name or description, study period, follow-up duration, number of participants or controls, number of cases, measurement of anthropometry (e.g. measured, self-reported), exposure (e.g. BMI, waist circumference), comparison (the contrast or metric of the exposure), RR, OR, HR and 95% CI, and adjustment for covariates. If several multivariable models were reported in the paper, the effect estimate most fully adjusted for potential confounders was used for the meta-analysis.

### Study quality assessment

Study quality of the included studies was assessed using the Newcastle–Ottawa scale^28^. Each study was appraised in terms of methodological quality categories: selection, comparability and outcome, with a total allowable score of up to 9 stars.

### Statistical analyses

Random effects models were used to calculate summary RRs and 95% CIs per 5 kg/m^2^ increase in BMI and per 10 cm increase in waist circumference^29^. The average of the natural logarithm of the RRs was estimated and the RR from each study was weighted using random effects weights^29^. Given the low prevalence of rheumatoid arthritis and the moderate effect sizes observed, different effect measures (ORs, HRs) were not converted, but used directly. If studies reported results by sex or other subgroups, but not overall, the subgroup-specific RR estimates were pooled with a fixed effects model before inclusion in the overall meta-analysis. To explore the dose–response relationship between increasing levels of adiposity and the risk of developing rheumatoid arthritis, linear and nonlinear dose–response analyses were conducted. For the linear dose–response analyses, the method of Greenland and Longnecker was used^30,31^. Study-specific linear dose–response slopes were calculated in a logit-linear model using the estimates across at least three categories of adiposity or by using continuous estimates directly. Fractional polynomial models were used to investigate potential nonlinear associations^32^. The extent of heterogeneity was assessed using the *I*^2^ statistic which describes the percentage of variability across studies in a meta-analysis, and *I*^2^ values of approximately 25%, 50% and 75% indicated low, moderate and high heterogeneity, respectively^33^. Publication bias was assessed with funnel plots^7,34^, the Egger linear regression test^35^, and the Begg rank correlation test^36^. If there was evidence of publication bias as indicated by *p* < 0.10 for Egger’s or Begg’s test or by asymmetry in the funnel plot, a sensitivity analysis was conducted to evaluate the potential impact of publication bias on the summary estimates by excluding outlying studies in the funnel plot from the analysis. To ensure that the results were not driven by one large study or a study with an extreme result, sensitivity analyses were conducted sequentially excluding each study from the analysis and assessing its impact on the summary estimate. Subgroup analyses were conducted to evaluate whether the results differed according to the following characteristics: sex, number of cases (< 250, 250–499, ≥ 500), geographic location (Europe, North America), measurement of BMI (measured, self-reported (validated), self-reported (not validated)), duration of follow-up (< 5 years, 5–9.9 years, 10–14.9 years 15–19.9 years, ≥ 20 years) and the extent of adjustment for confounding factors including age, education, social class, smoking, alcohol intake, physical activity, diabetes, and other comorbidities. All statistical analyses were performed using Stata/IC 13.1 (StataCorp LP, Texas, US).

## Results

### Study selection

**Figure 1:**
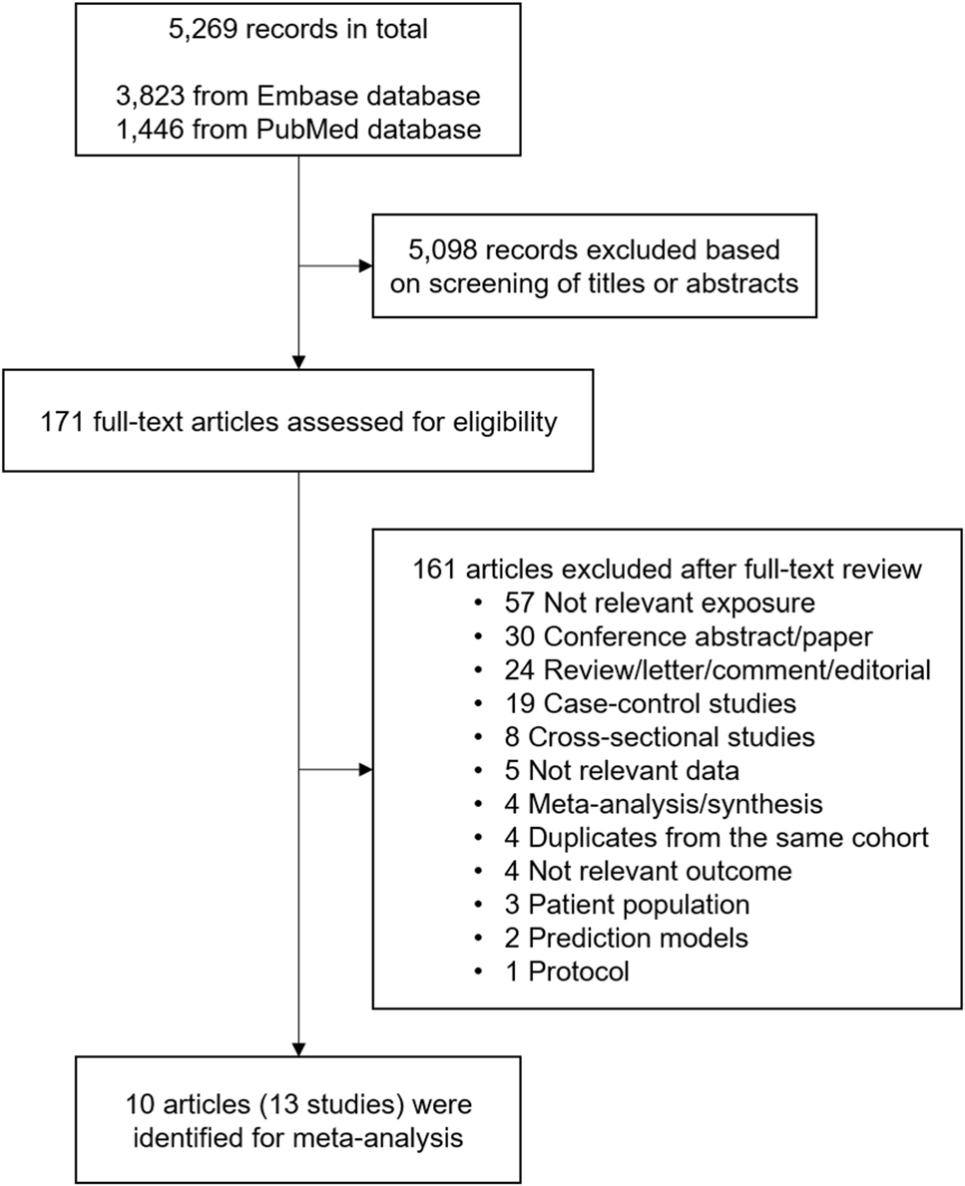
Flow diagram of study selection for the meta-analysis.

Figure 1 shows the flow diagram of study selection. A total of 5,269 records were identified from the Embase (3,823 records) and PubMed (1,446 records) databases by using index search terms. Based on screening the titles and abstracts, 171 potentially relevant articles were identified and evaluated for eligibility by assessing their full-texts. Ten articles including a total of 13 studies were included in the meta-analysis of adiposity and risk of rheumatoid arthritis.

### Study characteristics

The characteristics of included studies are summarised in Table 1. Among a total of 13 included studies, 8 studies were cohort studies^15–18,20,37,38^ and 5 studies (3 publications) were nested case–control studies within cohort studies^19,21,39^. Three publications included data from two studies each^15,19,39^, and one of these provided estimates from two different studies combined only^19^. While 8 studies (6 publications) included both men and women^17–19,21,38,39^, one study included men only^37^ and 4 studies (3 publications) were restricted to women only^15,16,20^. Eight studies (6 publications) performed anthropometric measures through a clinical examination^17–19,37–39^, while information on measures of adiposity was self-reported by participants in 4 studies (3 publications)^15,16,20^. In a nested case–control study using the UK General Practice Research Database (GPRD) conducted by Rodríguez et al*.*^21^, BMI was obtained from “physician-completed patient records”. To clarify this, we contacted the corresponding author of the study, as well as the Clinical Practice Research Datalink, who confirmed that although there is no way to be certain, it is reasonable to assume the anthropometric measurements were likely to have been performed by a physician or nurse at GP practices. Three studies (two publications, two risk estimates) reported results on waist circumference^18,19^, and three studies (two publications) reported on BMI in early adulthood (at age 18 years)^15,20^. Only one study reported on waist-to-hip ratio^20^, and body fat percentage^18^, and no studies reported results for hip circumference, thus analyses of these exposures were not possible. Most studies adjusted for potential confounders such as age, sex, smoking, and alcohol consumption. The average study quality score using the Newcastle–Ottawa scale^22,28^ was 8, with seven studies scoring 9 stars^17–19,37,39^, one study scoring 8 stars^21^, four studies scoring 7 stars^15,16,38^, and one study scoring 6 stars^20^ (Supplementary Table 1).

**Table 1:** Characteristics of included studies for the meta-analysis.

First author, publication year, country (ref. no)	Study name	Study period, follow-up duration	Number of participants, age, number of cases	Measurement of anthropometry	Exposure	Comparison	Relative risk, odds ratio or hazard ratio (95% CI)	Adjustment for covariates
Heliövaara M, 1993, Finland^37^	Social Insurance Institution's Mobile Clinic Health Examination Survey	1966/1972–1989, 18.1 years	28,364 men ≥ 15 years 119 cases	Measured	BMI	< 25.0 kg/m^2^ 25.0–29.9 ≥ 30.0	1.0 0.8 (0.5–1.2) 0.4 (0.2–1.2)	Age, smoking, geographical region, type of population, marital status, social class, perceived health
Cerhan JR, 2002, USA^20^	Iowa Women’s Health Study	1986–1997, 10.7 years	31,336 women 55–69 years 158 cases	Self-reported (validated)	BMI at baseline	< 23.4 kg/m^2^ 23.4–25.8 25.9–29.2 > 29.2	1.00 0.88 (0.56–1.37) 0.99 (0.64–1.53) 1.01 (0.65–1.56)	Age
					Waist-to-hip ratio at baseline	< 0.773 0.773–0.825 0.826–0.886 > 0.886	1.00 1.01 (0.67–1.54) 0.78 (0.49–1.22) 0.86 (0.55–1.34)	
					BMI at age 18	< 19.6 kg/m^2^ 19.6–21.1 21.2–22.9 > 22.9	1.00 1.16 (0.76–1.78) 0.87 (0.55–1.37) 0.97 (0.62–1.51)	
					BMI at age 30	< 21.2 kg/m^2^ 21.2–22.6 22.7–24.6 > 24.6	1.00 1.09 (0.71–1.69) 0.84 (0.53–1.33) 1.10 (0.71–1.69)	
					BMI at age 50	< 22.7 kg/m^2^ 22.7–24.8 24.9–27.5 > 27.5	1.00 0.90 (0.57–1.42) 1.15 (0.75–1.77) 0.96 (0.61–1.50)	
Rodríguez LAG, 2009, UK^21^	UK General Practice Research Database (GPRD)	1996–1997, ~ 2 years	Nested case–control study: 456 cases, 3,366 matched controls 20–79 years	Physician-completed patient records (Assumed to be measured)	BMI	< 20.0 kg/m^2^ 20.0–24.9 25.0–30.0 > 30.0	0.65 (0.43–0.98) 1.00 1.18 (0.94–1.98) 0.95 (0.68–1.34)	Age, sex, calendar year, number of referrals, and visits to a primary care physician in the previous year, smoking, alcohol intake, diabetes, cardiovascular disorders, other comorbidities, and pregnancy
Lu B, 2014, USA^15^	Nurses’ Health Study (NHS)	1976–2008, 25.2 years	109,896 women 30–55 years 826 cases	Self-reported (validated)	BMI	18.5–24.9 kg/m^2^ 25.0–29.9 ≥ 30.0	1.00 1.16 (0.99–1.35) 1.12 (0.92–1.37)	Age, community median income, smoking, alcohol use, and physical activity
					Cumulative average BMI	18.5–24.9 kg/m^2^ 25.0–29.9 ≥ 30.0	1.00 1.17 (1.00–1.37) 1.21 (0.97–1.50)	
					BMI at age 18	18.5–19.9 kg/m^2^ 20.0–22.9 23.0–24.9 ≥ 25.0	1.00 0.95 (0.80–1.12) 1.18 (0.93–1.48) 1.26 (0.99–1.61)	
Lu B, 2014, USA^15^	Nurses’ Health Study II (NHSII)	1989–2009, 17.8 years	108,727 women 25–42 years 355 cases	Self-reported (validated)	BMI	18.5–24.9 kg/m^2^ 25.0–29.9 ≥ 30.0	1.00 1.68 (1.30–2.17) 1.72 (1.31–2.45)	Age, community median income, smoking, alcohol use, and physical activity
					Cumulative average BMI	18.5–24.9 kg/m^2^ 25.0–29.9 ≥ 30.0	1.00 1.38 (1.08–1.77) 1.53 (1.16–2.01)	
					BMI at age 18	18.5–19.9 kg/m^2^ 20.0–22.9 23.0–24.9 ≥ 25.0	1.00 1.27 (0.99–1.62) 1.10 (0.76–1.61) 1.53 (1.10–2.14)	
Pahau H, 2014, Norway^38^	Nord-Trøndelag Health Study (HUNT)	1995/1997–2006/2008, ~ 11 years	31,568 participants ≥ 20 years 739 cases	Measured	BMI	Per 1 kg/m^2^ increase	1.04 (1.02–1.06)	Sex, age, current smoker, former smoker, hypertension, diabetes, previous cardiovascular disease
Harpsøe MC, 2014, Denmark^16^	Danish National Birth Cohort (DNBC)	1996/2002–2011, 11.4 years	75,008 pregnant women median age 30.2 years 315 cases	Self-reported (not validated)	BMI	< 18.5 kg/m^2^ 18.5–24.9 25.0–29.9 ≥ 30.0	0.82 (0.45–1.50) 1.00 1.12 (0.85–1.49) 1.53 (1.07–2.18)	Smoking, alcohol, parity, socio-occupational status
Lahiri M, 2014, UK^17^	European Prospective Investigation of Cancer-Norfolk	1993/1997–2010, 14.2 years	25,409 participants 40–79 years 138 cases	Measured	BMI	< 25.0 kg/m^2^ 25.0–29.9 ≥ 30.0	1.00 1.16 (0.78–1.74) 1.49 (0.91–2.42)	Age, gender, smoking, alcohol, occupational class, education, diabetes mellitus, parity, breast feeding
Ljung L, 2016, Sweden^19^	The Västerbotten Intervention Programme (VIP) and the Northern Sweden Multinational Monitoring of Trends and Determinants in Cardiovascular Disease (MONICA) project	1986/2013-NA, NA	Nested case–control study: 554 cases, 1,650 matched controls median age 51.9 years	Measured	BMI, all	< 25.0 kg/m^2^	1.00	Education and smoking, in case–control sets matched for age, sex and year of examination
						25.0–29.99	1.21 (0.96–1.52)	
						> = 30.0	1.45 (1.07–1.95)	
						Per 5 kg/m^2^ increase	1.13 (1.00–1.28)	
					Waist circumference (men/women)	≤ 102/ ≤ 88 cm > 102/ > 88 Per 1 cm increase	1.00 1.40 (0.92–2.14) 1.02 (1.01–1.04)	
Turesson C, 2016, Sweden^39^	The Malmö Diet and Cancer Study (MDCS)	1991/1996–2004, 10.5 years	Nested case–control study: 36/135 cases (men/ women), 144/537 matched controls mean age 58.5/57.9 years	Measured	BMI, men	18.5–25.0 kg/m^2^ 25.0–30.0 > 30.0 Per SD	1.00 0.44 (0.15–1.29) 0.14 (0.01–1.44) 0.58 (0.33–1.04)	Smoking, level of formal education and alcohol consumption, in case–control sets matched for sex and year of birth
					BMI, women	18.5–25.0 kg/m^2^ 25.0–30.0 > 30.0 Per SD	1.00 0.96 (0.57–1.61) 0.96 (0.48–1.92) 1.08 (0.86–1.36)	
Turesson C, 2016, Sweden^39^	The Malmö Preventive Medicine Program (MPMP)	1974/1992–2004, 21 years	Nested case–control study: 147/133 (men/ women) cases, 599/539 matched controls mean age 45.5/49.3 years	Measured	BMI, men	18.5–25.0 kg/m^2^ 25.0–30.0 > 30.0 Per SD	1.00 0.75 (0.46–1.17) 0.64 (0.20–2.02) 0.66 (0.41–1.07)	Smoking, level of formal education and alcohol consumption, in case–control sets matched for sex and year of birth
					BMI, women	18.5–25.0 25.0–30.0 > 30.0 Per SD	1.00 1.67 (0.94–2.69) 0.90 (0.36–2.26) 1.02 (0.78–1.33)	
Linauskas A, 2019, Denmark^18^	The Danish Diet, Cancer, and Health cohort	1993/1997–2016, 20.1 years	26,317 men and 28,720 women 50–64 years 210/456 (men/women) cases	Measured	BMI, men	18.5–24.99 25.0–29.99 ≥ 30.0 Per 1 kg/m^2^ increase	1.00 0.87 (0.65–1.18) 1.04 (0.69–1.57) 1.01 (0.98–1.05)	Age, smoking status and duration, socioeconomic status (education level), alcohol consumption, physical activity, and total intake of n − 3 fatty acids
					Waist circumference, men	Per 5 cm increase	1.03 (0.96–1.10)	
					Fat %, men	Per 5% increase	1.03 (0.90–1.17)	
					BMI, women	< 18.5 kg/m^2^ 18.5–24.99 25.0–29.99 ≥ 30.0 Per 1 kg/m^2^ increase	0.58 (0.19–1.83) 1.00 1.20 (0.98–1.48) 1.40 (1.08–1.83) 1.03 (1.01–1.05)	
					Waist circumference, women	Per 5 cm increase	1.05 (1.01–1.09)	
					Fat %, women	Per 5% increase	1.08 (1.01–1.16)	

*BMI* body mass index, *CI* confidence interval, *NA* not available.

### Body mass index and risk of rheumatoid arthritis

**Figure 2:**
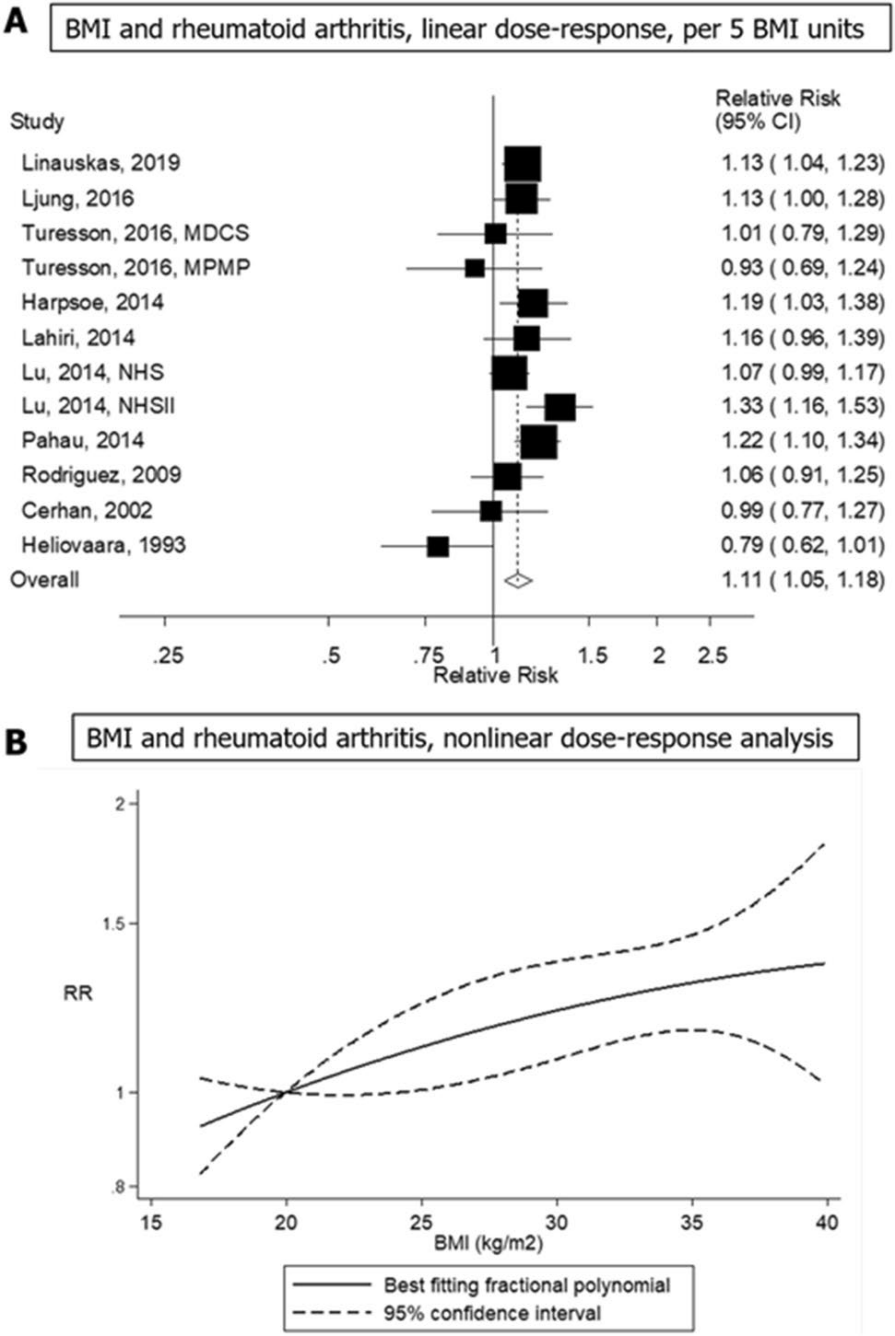
Linear and non-linear dose–response meta-analysis of body mass index and risk of rheumatoid arthritis, per 5 kg/m^2^.

Thirteen studies (ten publications, twelve risk estimates)^15–21,37–39^, involving a total of 473,641 participants and 4,777 cases were included in the linear dose–response meta-analysis of BMI and rheumatoid arthritis, and the summary RR per 5 kg/m^2^ increase in BMI was 1.11 (95% CI 1.05–1.18, *I*^2^ = 50%; Fig. 2A). The funnel plot (Supplementary Fig. 1) and Begg's test (*P* = 0.09) showed some suggestion of publication bias, but not Egger's test (*P* = 0.14). When excluding one outlying study (Heliövaara et al*.*^37^), the summary RR per 5 kg/m^2^ increase in BMI was 1.14 (95% CI 1.08–1.19), the heterogeneity was reduced (*I*^2^ = 26%, *P* = 0.20; Supplementary Fig. 2), and the *P* values for Begg’s test and Egger’s test were 0.21 and 0.37, respectively. In sensitivity analyses excluding one study at a time from the meta-analysis, the summary RR per 5 kg/m^2^ increase in BMI ranged from 1.10 (95% CI 1.04–1.16, *I*^2^ = 38%) when the NHSII study by Lu et al*.*^15^ was excluded, to 1.14 (95% CI 1.08–1.19, *I*^2^ = 26%) when the study by Heliövaara et al*.*^37^ was excluded (Supplementary Fig. 3). Twelve studies (nine publications, 442,073 participants and 4,038 cases) were included in the nonlinear dose–response analysis, which revealed evidence of a positive dose–response relationship and no indication of a nonlinear association (*P*_nonlinearity_ = 0.56) (Fig. 2B).

**Figure 3:**
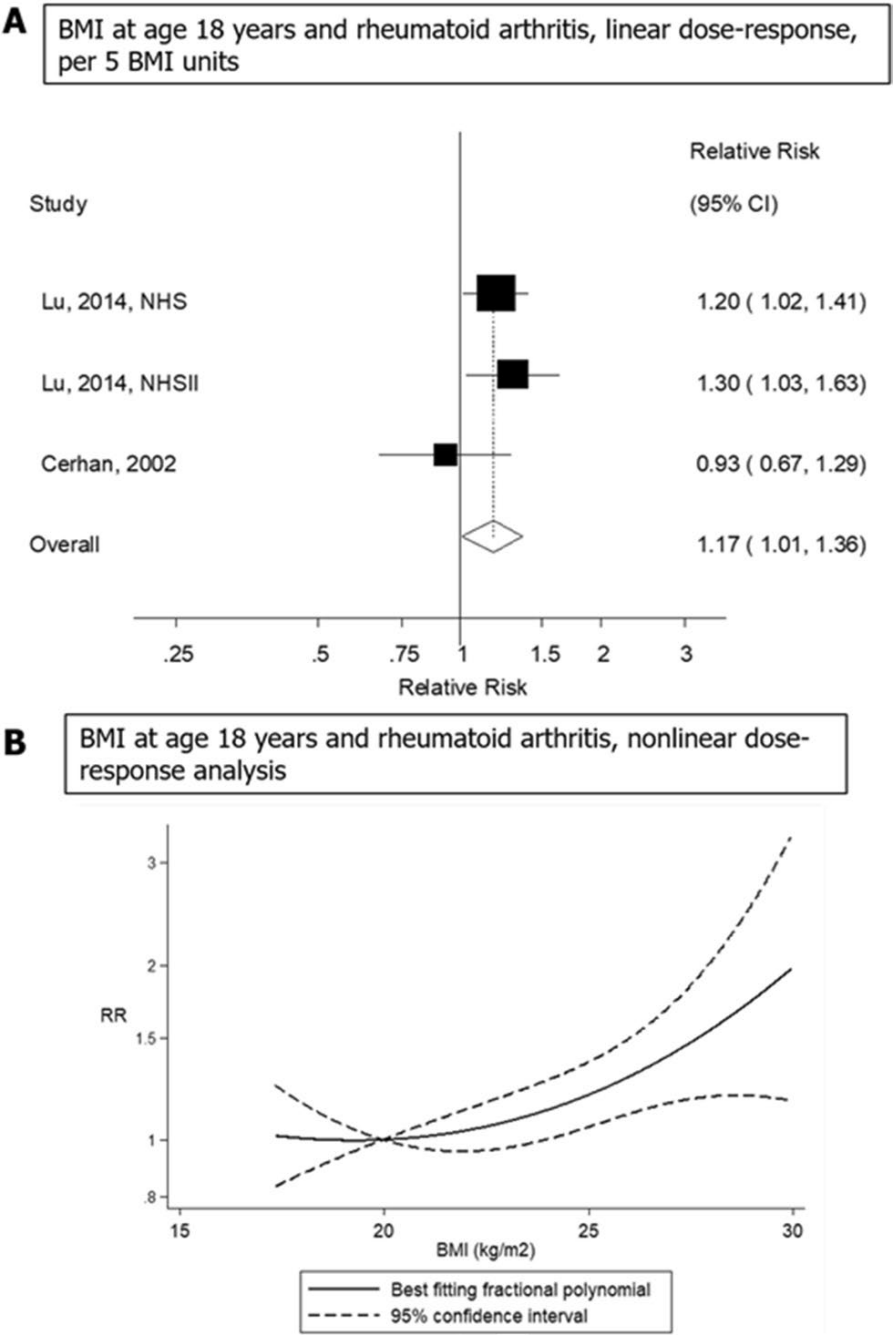
Linear and non-linear dose–response meta-analysis of body mass index in early adulthood (at age 18 years) and risk of rheumatoid arthritis, per 5 kg/m^2^.

### Body mass index in early adulthood and risk of rheumatoid arthritis

Three cohort studies (two publications, 249,959 participants and 1,263 cases)^15,20^ were included in the analysis of BMI in early adulthood (at age 18 years) and risk of developing rheumatoid arthritis. The summary RR per 5 kg/m^2^ increase in BMI in early adulthood (at age 18 years) was 1.17 (95% CI 1.01–1.36, *I*^2^ = 26%; Fig. 3A). There was no evidence of publication bias by inspection of the funnel plot (Supplementary Fig. 4), or with Egger's test (*P* = 0.99) or Begg’s test (*P* = 0.57), although the number of studies was limited. There was no evidence of a nonlinear association between BMI in early adulthood (at age 18 years) and the risk of rheumatoid arthritis (*P*_nonlinearity_ = 0.99) (Fig. 3B).

**Figure 4:**
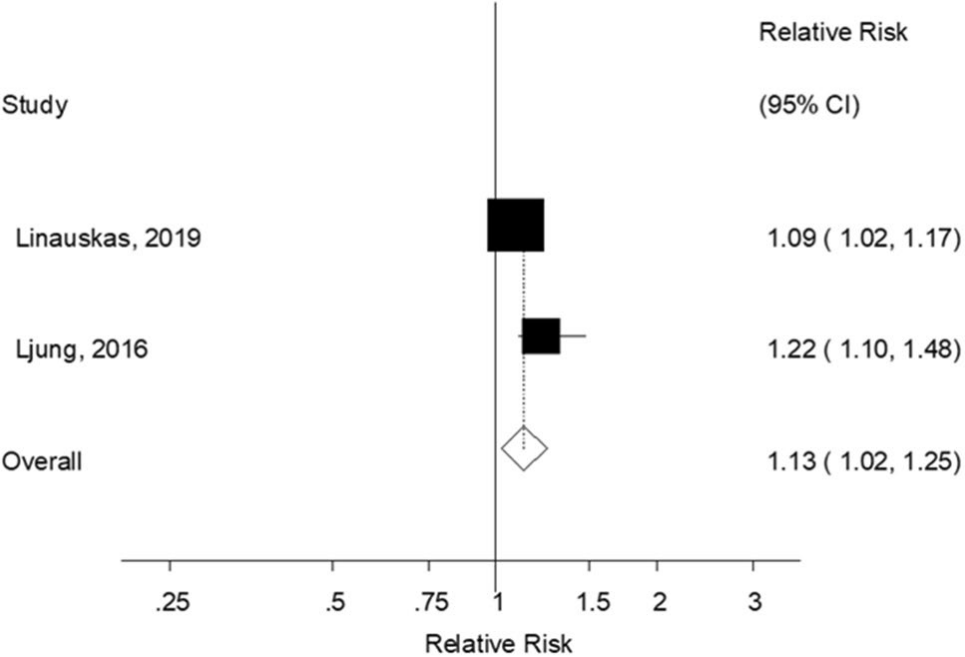
Linear dose–response meta-analysis of waist circumference and risk of rheumatoid arthritis, per 10 cm.

### Waist circumference and risk of rheumatoid arthritis

Three prospective studies (two publications, two risk estimates, 55,584 participants and 804 cases)^18,19^ were included in the meta-analysis of waist circumference and risk of rheumatoid arthritis. The summary RR per 10 cm increase in waist circumference was 1.13 (95% CI 1.02–1.25, *I*^2^ = 44%; Fig. 4). Nonlinear dose–response analyses were not possible because both publications reported results on a continuous scale.

### Subgroup analyses

**Figure 5:**
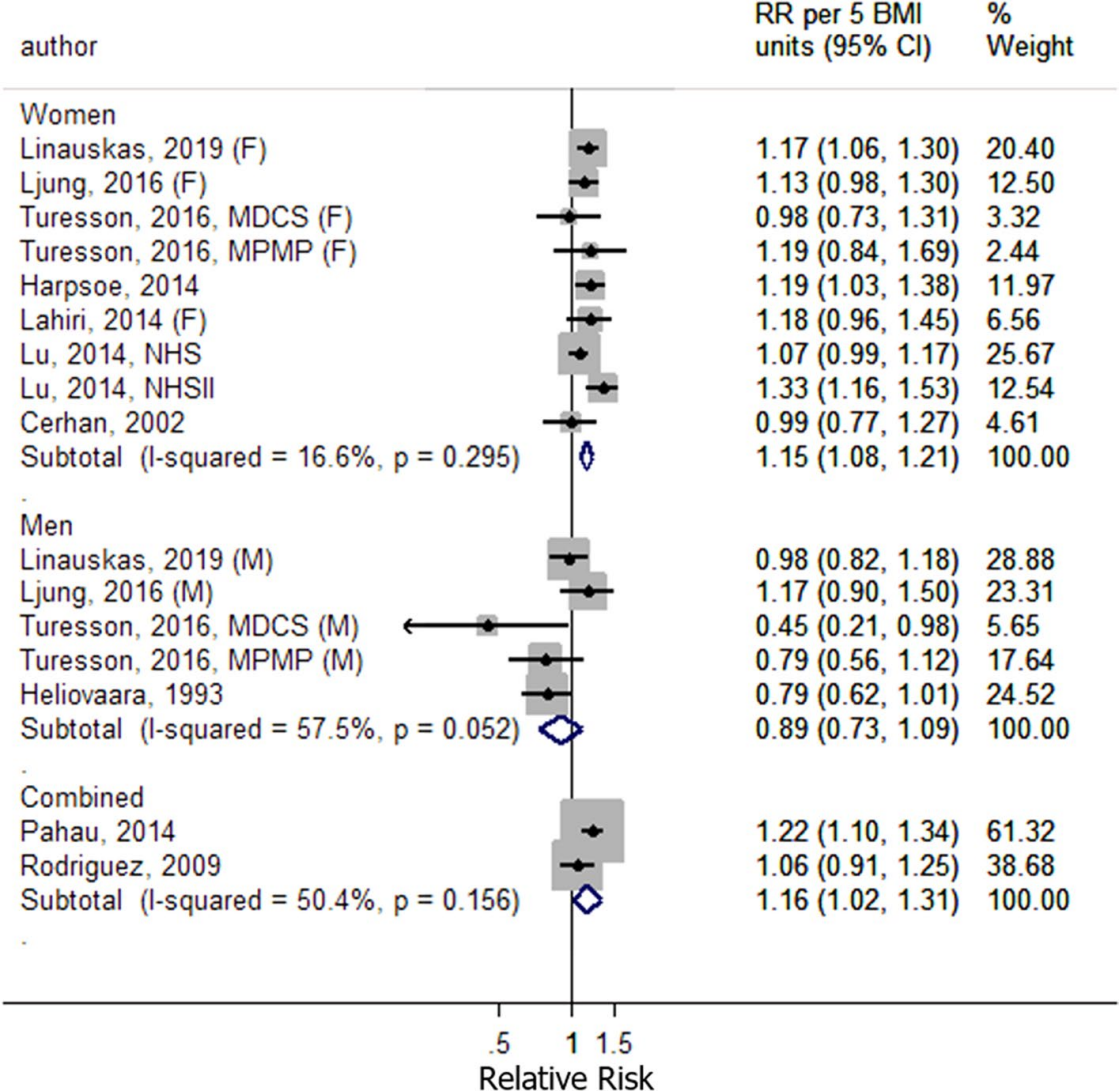
Sex-specific subgroup analyses for the linear dose–response meta-analysis of body mass index and risk of rheumatoid arthritis.

To investigate potential heterogeneity in the results, subgroup analyses were conducted stratified by sex, geographic location, number of cases, BMI measurement, duration of follow-up and adjustment for confounding factors. There was evidence of heterogeneity by sex (*P* = 0.02), with a positive association observed in women 1.15 (95% CI 1.08–1.21, *I*^2^ = 17%, Fig. 5), but not in men 0.89 (95% CI 0.73–1.09, *I*^2^ = 58%; Fig. 5) (Table 2). The remaining subgroup analyses showed little evidence of heterogeneity between subgroups (Table 2).

**Table 2:** Subgroup analyses for the linear dose–response meta-analysis of body mass index and risk of rheumatoid arthritis.

Study groups	n	RR	(95% CI)	*I*^2^ (%)	*P* value for heterogeneity	
					Within each subgroup	Between subgroups
All studies	12	1.11	(1.05–1.18)	50.2	0.002	
**Sex**						0.06
Men	5	0.89	(0.73–1.09)	57.5	0.05	
Women	9	1.15	(1.08–1.21)	16.6	0.30	
Combined	2	1.16	(1.02–1.31)	50.4	0.16	
**Geographic location**						0.65
Europe	9	1.10	(1.03–1.18)	44.7	0.07	
North America	3	1.14	(0.96–1.34)	73.8	0.02	
**Number of cases**						0.22
< 250	4	0.99	(0.84–1.16)	50.0	0.11	
250–499	4	1.15	(1.01–1.31)	58.2	0.07	
≥ 500	4	1.13	(1.08–1.19)	16.3	0.31	
**BMI measurement**						0.41
Measured	8	1.09	(1.01–1.18)	49.5	0.05	
Self-reported (validated)	3	1.14	(0.96–1.34)	73.8	0.02	
Self-reported (not validated)	1	1.19	(1.03–1.38)	–	–	
**Duration of follow-up**						0.99
< 5 years	1	1.06	(0.91–1.25)	–	–	
5–9.9 years	1	1.13	(1.00–1.28)	–	–	
10–14.9 years	4	1.14	(1.04–1.26)	19.2	0.29	
15–19.9 years	2	1.04	(0.62–1.72)	92.6	< 0.0001	
≥ 20 years	4	1.11	(1.05–1.17)	4.4	0.37	
**Adjustment for confounders**						
**Age**						0.55
Yes	11	1.10	(1.03–1.18)	53.5	0.02	
No	1	1.19	(1.03–1.38)	–	–	
**Education**						0.87
Yes	4	1.13	(1.06–1.20)	0.0	0.84	
No	8	1.10	(1.00–1.20)	67.0	0.003	
**Social class**						0.91
Yes	6	1.09	(0.97–1.24)	70.9	0.004	
No	6	1.14	(1.08–1.19)	0.0	0.46	
**Smoking**						0.49
Yes	11	1.12	(1.05–1.19)	52.5	0.02	
No	1	0.99	(0.77–1.27)	–	–	
**Alcohol intake**						0.33
Yes	7	1.14	(1.07–1.21)	29.1	0.21	
No	5	1.03	(0.90–1.19)	70.4	0.009	
**Physical activity**						0.37
Yes	3	1.16	(1.04–1.29)	69.4	0.04	
No	9	1.08	(1.00–1.17)	48.0	0.05	
**Diabetes**						0.52
Yes	3	1.17	(1.08–1.26)	1.7	0.36	
No	9	1.09	(1.01–1.18)	57.2	0.02	
**Other comorbidities**						0.61
Yes	2	1.16	(1.02–1.31)	50.4	0.16	
No	10	1.10	(1.03–1.18)	52.3	0.03	

n refers to number of risk estimates (one publication reported results for two studies combined so the total number of studies is 13).

## Discussion

In this meta-analysis, higher BMI in middle age, BMI in early adulthood, and waist circumference were associated with an increased risk of rheumatoid arthritis. There was no evidence of nonlinearity, and positive dose–response relationships were apparent for both BMI in middle age and BMI in early adulthood. The positive association between BMI in middle age and rheumatoid arthritis was restricted to women, and no clear association was observed in men; this could be due to an interaction with hormone-related factors or other sex-specific exposures, which may contribute to the higher prevalence of rheumatoid arthritis in women^26^. It has also been reported that differences in body fat distribution between men and women may contribute to sex differences in the risk of rheumatoid arthritis^27^. Due to a lack of data from primary studies, it was not possible to evaluate the sex-specific associations for waist circumference, and further research is needed to determine whether the sex difference in association is only for BMI, or also for other measures of adiposity.

The current findings are consistent with a previous meta-analysis, which also found increased risk of rheumatoid arthritis with increasing BMI^22,23^. Feng et al*.*^23^, reported a summary RR for every 5 kg/m^2^ increase in BMI of 1.13 (95% CI 1.01–1.26, *I*^2^ = 83.0%) based on data from 5 cohort studies and 6 case–control studies. The heterogeneity between studies was much lower in the current meta-analysis than in the previous meta-analysis, and this is probably to a large extent because we only included cohort studies. This avoids recall bias and reduces the possibility of selection bias and reverse causation. The larger number of cohort studies in the present analysis contributed to more precise risk estimates, and enabled assessment of associations of other adiposity measures, such as waist circumference and BMI in early adulthood, with risk of rheumatoid arthritis. Some studies have suggested that bariatric surgery may improve symptoms in patients with rheumatoid arthritis^40,41^, however, a recent Swedish study found no significant association between bariatric surgery and the risk of developing rheumatoid arthritis (HR = 0.92, 95% CI 0.59–1.46), but statistical power to detect a moderate association may have been low and confidence intervals were wide^42^.

The finding that high BMI in early adulthood (at age 18 years) was associated with an increased risk of rheumatoid arthritis might suggest that early life risk factors may be of importance in the aetiology of rheumatoid arthritis^43,44^. In a review by Colebatch et al*.*^43^, the authors summarised evidence on the effect of early life environmental factors on the risk of future rheumatoid arthritis. Among the findings, it was reported that early initiation of breastfeeding may be associated with a reduced risk of rheumatoid arthritis^45^, while high birth weight^45,46^ and maternal smoking in pregnancy^47^ may contribute to an increased risk of rheumatoid arthritis. On the other hand, high BMI in early adulthood is correlated with high BMI in middle age^48^, and it is possible that longer duration of obesity may confer an even greater risk of rheumatoid arthritis.

With regard to the underlying mechanisms that could contribute to the observed associations, it is known that adipose tissue plays an important role in the inflammatory process^49^. This is supported by evidence that adipocytes produce not only adipocytokines but also inflammatory cytokines^49^. Thus, adiposity can increase systemic inflammation, leading to increased susceptibility for developing rheumatoid arthritis.

There are several strengths of this study. The current meta-analysis had 2–3 times as many cohort studies as three meta-analyses published in 2015, 2016 and 2018^22–24^, and we identified two additional cohort studies^18,38^ that were not identified in the most recently published meta-analysis in 2019^50^. The inclusion of additional studies and a large total number of cases (n = 4,777) and nearly 0.5 million participants provided sufficient statistical power to detect moderate associations and contributed to more robust results. Another major advantage of the current meta-analysis was that it only included prospective cohort studies. While the previous meta-analyses included many retrospective case–control studies that may have been affected by recall and selection biases^22–24,50^, the current meta-analysis is less likely to have been affected by such biases. Detailed dose–response analyses were conducted to clarify the dose–response relationship between adiposity and rheumatoid arthritis, and detailed subgroup analyses were conducted to investigate sources of heterogeneity. A novelty of this study was the analyses of additional measures of adiposity, specifically waist circumference and BMI in early adulthood, with risk of rheumatoid arthritis. BMI is considered to be a useful indicator of obesity, but this index does not reflect abdominal body fat distribution, and the accuracy of the index can vary according to age, sex and body composition^51^. When developing prevention interventions for rheumatoid arthritis, it may be informative to assess the association between several other measures of adiposity and the risk of developing rheumatoid arthritis.

There are several limitations of this study that should be noted. In some of the included studies anthropometric measurements were self-reported. Consequently, the results might have been influenced by misclassification of the exposure. However, nine of the thirteen included studies performed anthropometric measurements through a clinical examination^17–19,21,37–39^, and showed similar results to the overall summary estimate. Only four studies used self-reported data^15,16,20^. There was no heterogeneity in associations in subgroup analyses stratified by whether anthropometric measures were measured or self-reported.

Heterogeneity between studies, publication bias and confounding are potentially important limitations of any meta-analysis. Some degree of heterogeneity is expected because studies may differ with regard to the assessment of adiposity, other risk factors, the outcome, underlying risk factor profile and disease rates, as well as analytical approaches including the extent of adjustment for confounding factors. Sources of heterogeneity were investigated in subgroup analyses, however, with the exception of the difference in association for men and women, there was little evidence of heterogeneity between other subgroups including geographic location, number of cases, measurement of BMI, duration of follow-up, and adjustment for confounding factors (including education, social class, smoking, alcohol, physical activity, diabetes or other co-morbidities). Although most of the individual included studies adjusted for important potential confounders, it is important to note that residual and unmeasured confounding is possible. For example, one study suggested that consumption of sugar-sweetened beverages may increase risk of rheumatoid arthritis^52^ and other studies have found sugar-sweetened beverages to increase weight gain^53^. Given that little is known with certainty about diet and rheumatoid arthritis, and most studies have not adjusted for dietary factors, we cannot exclude the possibility that the observed association partly could be due to confounding by sugar-sweetened beverages or other dietary factors. Although there was some indication of publication bias in the analysis of BMI, this appeared to be explained by one outlying study, which when excluded did not substantially alter the results, but which also appeared to explain a large part of the heterogeneity between studies. Thus, it seems less likely that publication bias would have substantially impacted the overall conclusions. Another limitation was that the study populations were limited to North American and European populations and therefore had limited geographical coverage. Therefore, further research is needed to evaluate the association between adiposity and the risk of rheumatoid arthritis in other regions, including Africa, South America and Asia–Pacific.

According to the Global Burden of Disease 2010 study, the number of people living with rheumatoid arthritis is expected to increase considerably over the coming decades, especially in low-income and middle-income countries^25^. In addition, overweight and obesity have become a considerable problem in most regions of the world^54^, including low-income and middle-income countries such as China, Brazil and Indonesia as well as in high-income countries^55^.

As the number of studies on adiposity variables other than BMI was limited, further studies are needed on a range of alternative adiposity measures including waist circumference, hip circumference, waist-to-hip ratio, waist-to-height ratio, BMI in early adulthood, and weight change throughout the life course, and to clarify whether the sex-specific results for BMI also apply to these other measures of adiposity. Because excess weight to a large degree is driven by diet and lifestyle factors^56^, it is possible that certain lifestyle-related risk factors (e.g. consumption of sugar-sweetened soft drinks and low physical activity) may contribute to the development of rheumatoid arthritis partly through an effect on adiposity^52,57–59^, and further studies are needed to clarify if this is the case.

## Conclusion

This study confirms a positive association between levels of adiposity and the risk of developing rheumatoid arthritis. Higher BMI in middle age, BMI in early adulthood (at age 18 years), and waist circumference were associated with a higher risk of rheumatoid arthritis in dose–response meta-analyses. The current analysis suggests that excess weight may be a risk factor for rheumatoid arthritis in women, but further cohort studies are needed to clarify sex-specific associations with adiposity measures other than BMI as the available data is limited. Since most of the existing studies are from Europe and the US, additional studies are needed from other geographic regions as well. Based on these findings, prevention strategies targeting weight reduction and maintenance of a healthy weight may contribute to reducing the burden rheumatoid arthritis places on society.

## Supplementary information

Supplementary information.

## Data Availability

The datasets generated and/or analysed during the current study are available from the corresponding author on reasonable request.
