# Supplementary material for: Adiposity and the risk of rheumatoid arthritis: a systematic review and meta-analysis of cohort studies
Source: Sci Rep. 2020 Sep 29;10:16006. doi: 10.1038/s41598-020-71676-6 (PMC7524740; doi:10.1038/s41598-020-71676-6)
Supplement: Supplementary file 1 — Supplementary information. [file 41598_2020_71676_MOESM1_ESM.docx]

Adiposity and the risk of rheumatoid arthritis: a systematic review and meta-analysis of cohort studies

Tomoya Ohno^1,2^, Dagfinn Aune^1,3,4^†, Alicia K. Heath^1^†*

†Shared last authors

^1^ Department of Epidemiology and Biostatistics, School of Public Health, Imperial College London, London, United Kingdom

^2^Present address: Oncology division, Novartis Pharma K.K., Tokyo, Japan

^3^Department of Nutrition, Bjørknes University College, Oslo, Norway

^4^Department of Endocrinology, Morbid Obesity and Preventive Medicine, Oslo University Hospital, Oslo, Norway

*Correspondence to: Dr. Dagfinn Aune, Department of Epidemiology and Biostatistics,

School of Public Health, Imperial College London, St. Mary's Campus, Norfolk Place, Paddington, London W2 1PG, UK.

Telephone: +44 (0) 20 7594 8478

E-mail: d.aune@imperial.ac.uk

**Supplementary Table 1. Study quality assessment of included studies according to the NEWCASTLE-OTTAWA scale**

| **Study** | | **Selection** | | | | **Comparability** | **Outcome** | | | **Total** |
| --- | --- | --- | --- | --- | --- | --- | --- | --- | --- | --- |
| **First author, publication year, study design** | **Study name or description** | **Representativeness of the exposed cohort** | **Selection of the non-exposed cohort** | **Ascertainment of exposure** | **Demonstration that outcome of interest was not present at start of study** | **Comparability of cohorts on the basis of the design or analysis** | **Assessment of outcome** | **Was follow-up long enough for outcome to occur**** | **Adequacy of follow-up of cohorts** | **A maximum of 9 stars** |
| Heliövaara M, 1993, cohort study (38) | Social Insurance Institution's Mobile Clinic Health Examination Survey | ★ | ★ | ★ | ★ | ★★ | ★ | ★ | ★ | **9** |
| Cerhan JR, 2002, cohort study (20) | Iowa Women’s Health Study | - | ★ | - | ★ | ★ | ★ | ★ | ★ | **6** |
| Rodríguez LA, 2009, nested case-control study* (21) | UK General Practice Research Database (GPRD) | ★ | ★ | ★ | ★ | ★★ | ★ | - | ★ | **8** |
| Lu B, 2014, US, cohort study (15) | Nurses’ Health Study (NHS) | - | ★ | - | ★ | ★★ | ★ | ★ | ★ | **7** |
|  | Nurses’ Health Study II (NHSII) | - | ★ | - | ★ | ★★ | ★ | ★ | ★ | **7** |
| Pahau H, 2014, cohort study (39) | Nord-Trøndelag Health Study (HUNT) | ★ | ★ | ★ | ★ | ★★ | - | ★ | - | **7** |
| Harpsøe MC, 2014, cohort study (16) | Danish National Birth Cohort (DNBC) | - | ★ | - | ★ | ★★ | ★ | ★ | ★ | **7** |
| Lahiri M, 2014, cohort study (17) | European Prospective Investigation of Cancer-Norfolk and the Norfolk Arthritis Register (EPIC-2-NOAR Study) | ★ | ★ | ★ | ★ | ★★ | ★ | ★ | ★ | **9** |
| Ljung L, 2016, nested case-control study* (19) | The Västerbotten Intervention Programme (VIP) and the Northern Sweden Multinational Monitoring of Trends and Determinants in Cardiovascular Disease (MONICA) project | ★ | ★ | ★ | ★ | ★★ | ★ | ★ | ★ | **9** |
| Turesson C, 2016, nested case-control study* (40) | The Malmö Diet Cancer Study (MDCS) | ★ | ★ | ★ | ★ | ★★ | ★ | ★ | ★ | **9** |
|  | The Malmö Preventive Medicine Program (MPMP) | ★ | ★ | ★ | ★ | ★★ | ★ | ★ | ★ | **9** |
| Linauskas A, 2019, cohort study (18) | The Danish Diet, Cancer, and Health cohort and the Danish National Patient Registry | ★ | ★ | ★ | ★ | ★★ | ★ | ★ | ★ | **9** |

*For nested case-control studies, allocation of stars was conducted using the same scoring as for cohort studies.

**A follow-up period of 5 years was selected as long enough for assessing the relationship between adiposity and rheumatoid arthritis.

**Supplementary Figure 1. Funnel plot for the linear dose-response meta-analysis of body mass index and risk of rheumatoid arthritis**

|  |
| --- |
| 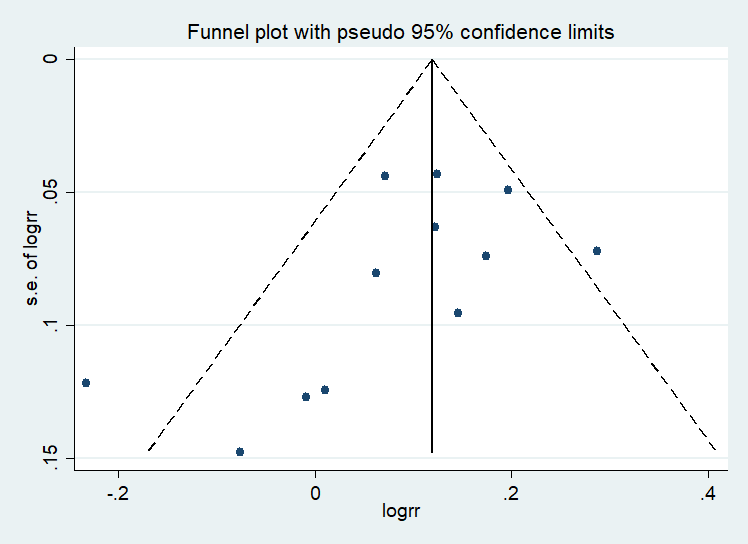 |
|  |

**Supplementary Figure 2. Linear dose-response meta-analysis of body mass index and risk of rheumatoid arthritis when excluding the study by Heliövaara *et al*, per 5 kg/m^2^**

|  |
| --- |
| 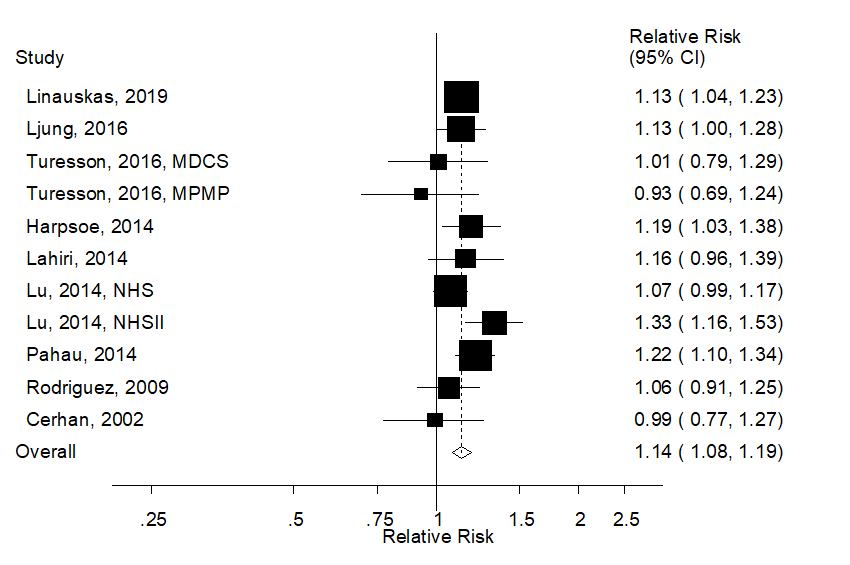 |
| *I^2^* = 26.0%, *P* = 0.20 |

**Supplementary Figure 3. Linear dose-response meta-analysis of body mass index and risk of rheumatoid arthritis when excluding each single study**

|  |
| --- |
| 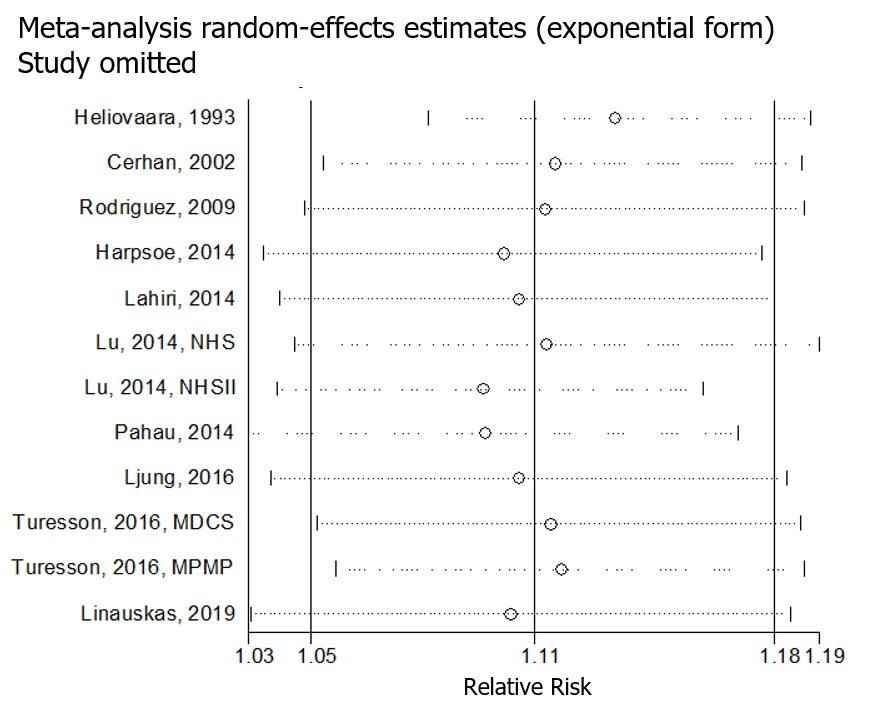 |
|  |
| \| **Study omitted** \| **RR** \| **[95% CI]** \| \| \| ***I^2^* (%)** \| ***P* value for heterogeneity** \| \| --- \| --- \| --- \| --- \| --- \| --- \| --- \| \| Heliövaara, 1993 \| 1.14 \| 1.08 \| to \| 1.19 \| 26.0 \| 0.20 \| \| Cerhan, 2002 \| 1.12 \| 1.05 \| to \| 1.19 \| 52.5 \| 0.02 \| \| Lu, 2014, NHS \| 1.12 \| 1.04 \| to \| 1.19 \| 51.5 \| 0.02 \| \| Lu, 2014, NHSII \| 1.10 \| 1.04 \| to \| 1.16 \| 38.3 \| 0.09 \| \| Harpsøe, 2014 \| 1.10 \| 1.03 \| to \| 1.18 \| 53.5 \| 0.02 \| \| Lahiri, 2014 \| 1.11 \| 1.04 \| to \| 1.18 \| 54.6 \| 0.02 \| \| Linauskas, 2019 \| 1.11 \| 1.03 \| to \| 1.19 \| 54.7 \| 0.02 \| \| Rodriguez, 2009 \| 1.12 \| 1.05 \| to \| 1.19 \| 53.6 \| 0.02 \| \| Ljung, 2016 \| 1.11 \| 1.04 \| to \| 1.18 \| 54.7 \| 0.02 \| \| Turesson, 2016, MDCS \| 1.12 \| 1.05 \| to \| 1.19 \| 53.1 \| 0.02 \| \| Turesson, 2016, MPMP \| 1.12 \| 1.06 \| to \| 1.19 \| 50.8 \| 0.03 \| \| Pahau, 2014 \| 1.10 \| 1.03 \| to \| 1.17 \| 47.8 \| 0.04 \| |

**Supplementary Figure 4. Funnel plot for the linear dose-response meta-analysis of body mass index in early adulthood (at age 18 years) and risk of rheumatoid arthritis**

|  |
| --- |
| 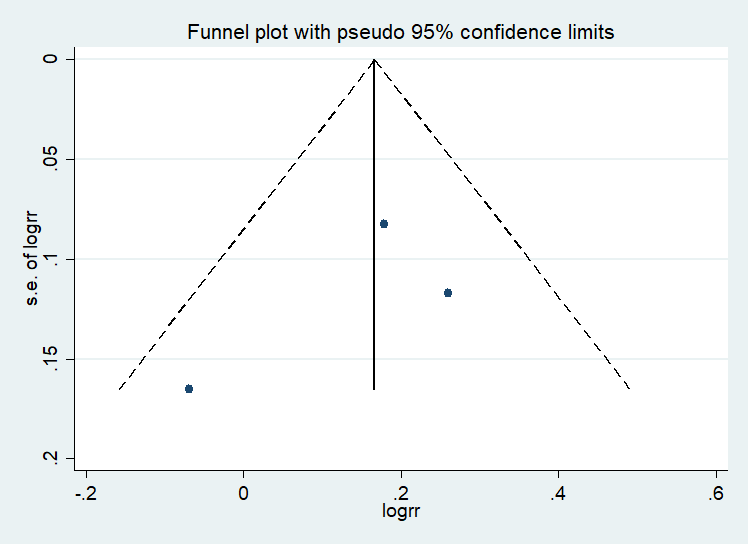 |
|  |
